# Supplementary material for: Understanding pre-training data effects in retinal foundation models using two large fundus cohorts
Source: Nat Commun. 2026 Feb 28;17:3309. doi: 10.1038/s41467-026-70077-z (PMC13065816; doi:10.1038/s41467-026-70077-z)
Supplement: Supplementary file 1 — Supplementary Information [file 41467_2026_70077_MOESM1_ESM.pdf]

Supplementary Information

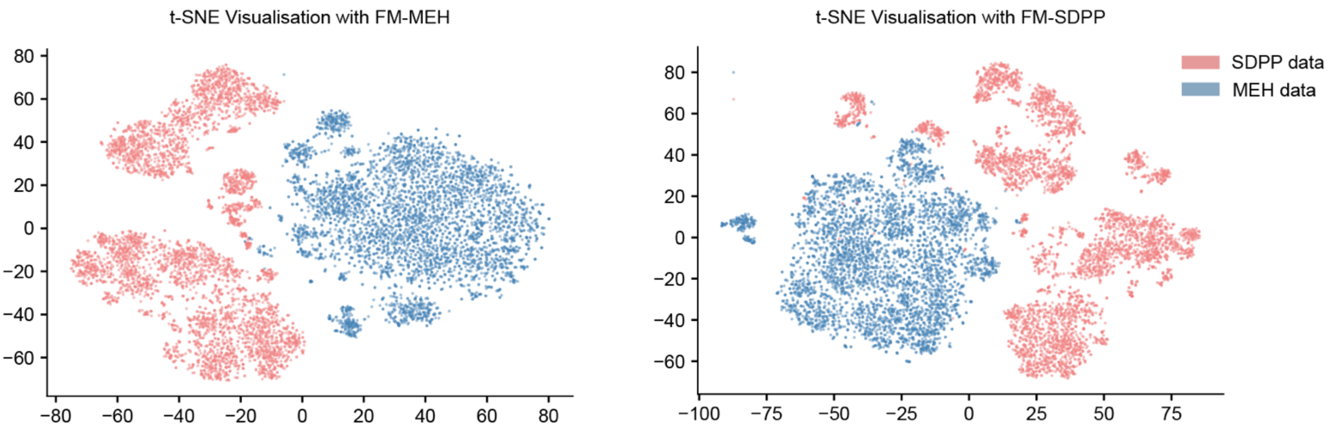

Supplementary Figure 1. t-SNE visualisation for MEH and SDPP data (5000 randomly sampled data points), respectively with features extracted by foundation models developed in each site (FM-MEH and FM-SDPP) with DINOV2.

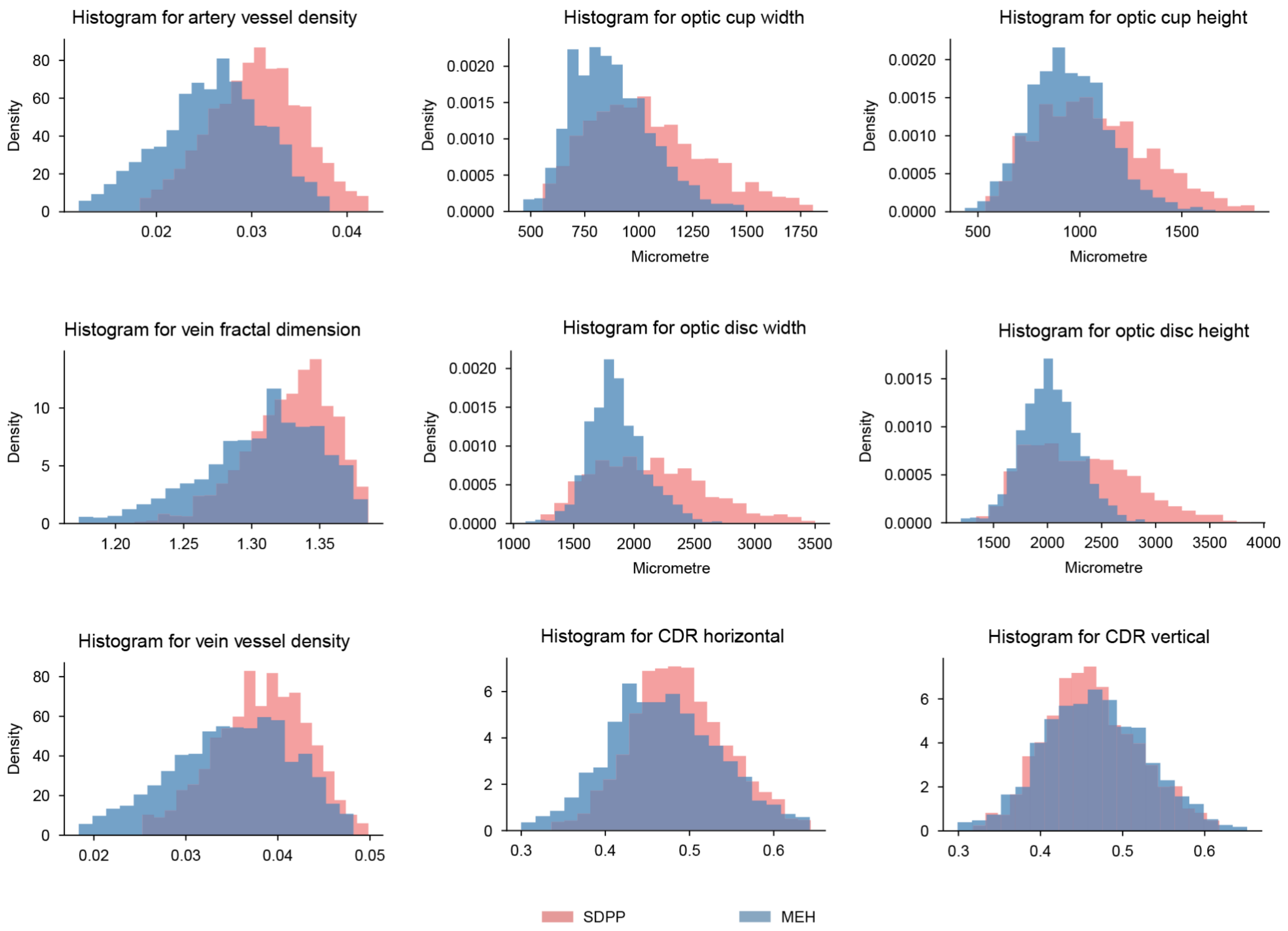

Supplementary Figure 2. Extra clinical meaningful morphological indices of MEH and SDPP data (5000 randomly sampled data points), obtained with AutoMorph. These demonstrate the distinct distribution of data from Moorfields Eye Hospital and SDPP. CDR indicates the optic cup-to-disc ratio. CDR horizontal calculates the ratio of width between the optic cup to the disc while CDR vertical for height ratio.

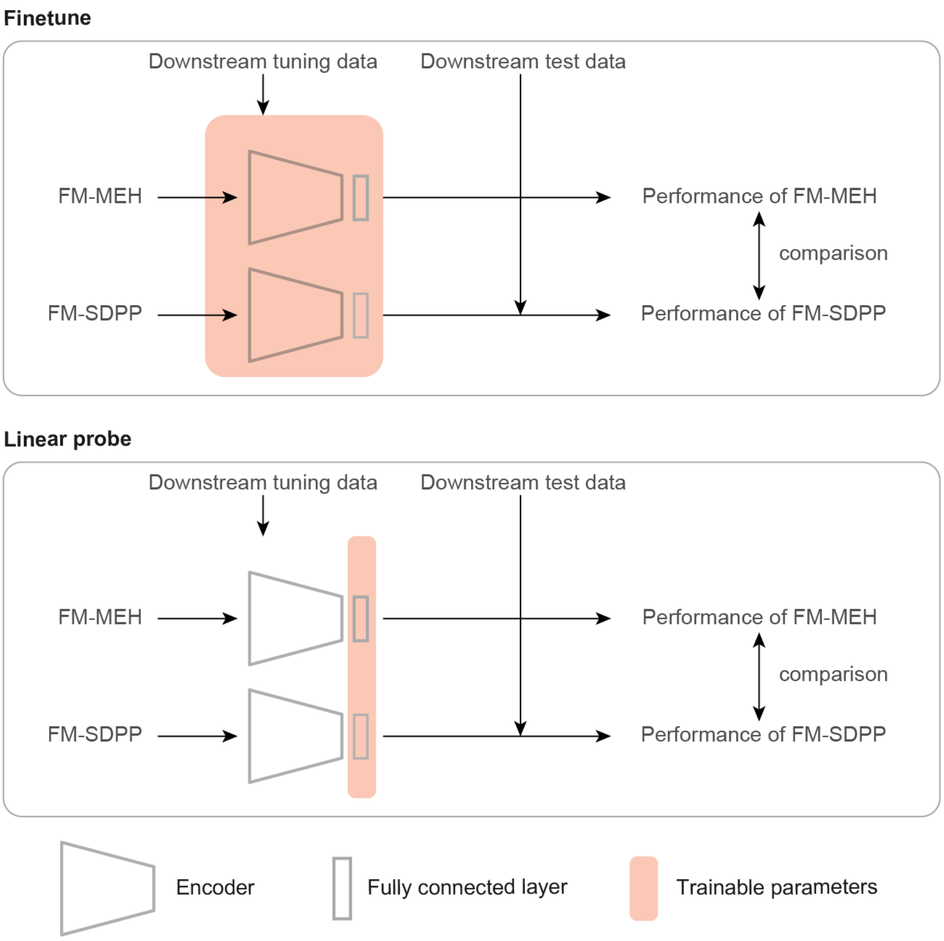

Supplementary Figure 3. Schematic diagram of foundation model adaptations to downstream tasks. Fine-tuning tunes the encoder and fully connected layer using downstream fine-tuning data. Linear probe tunes the fully connected layer only. The performances of FM-MEH and FM-SDPP on downstream test data are compared.

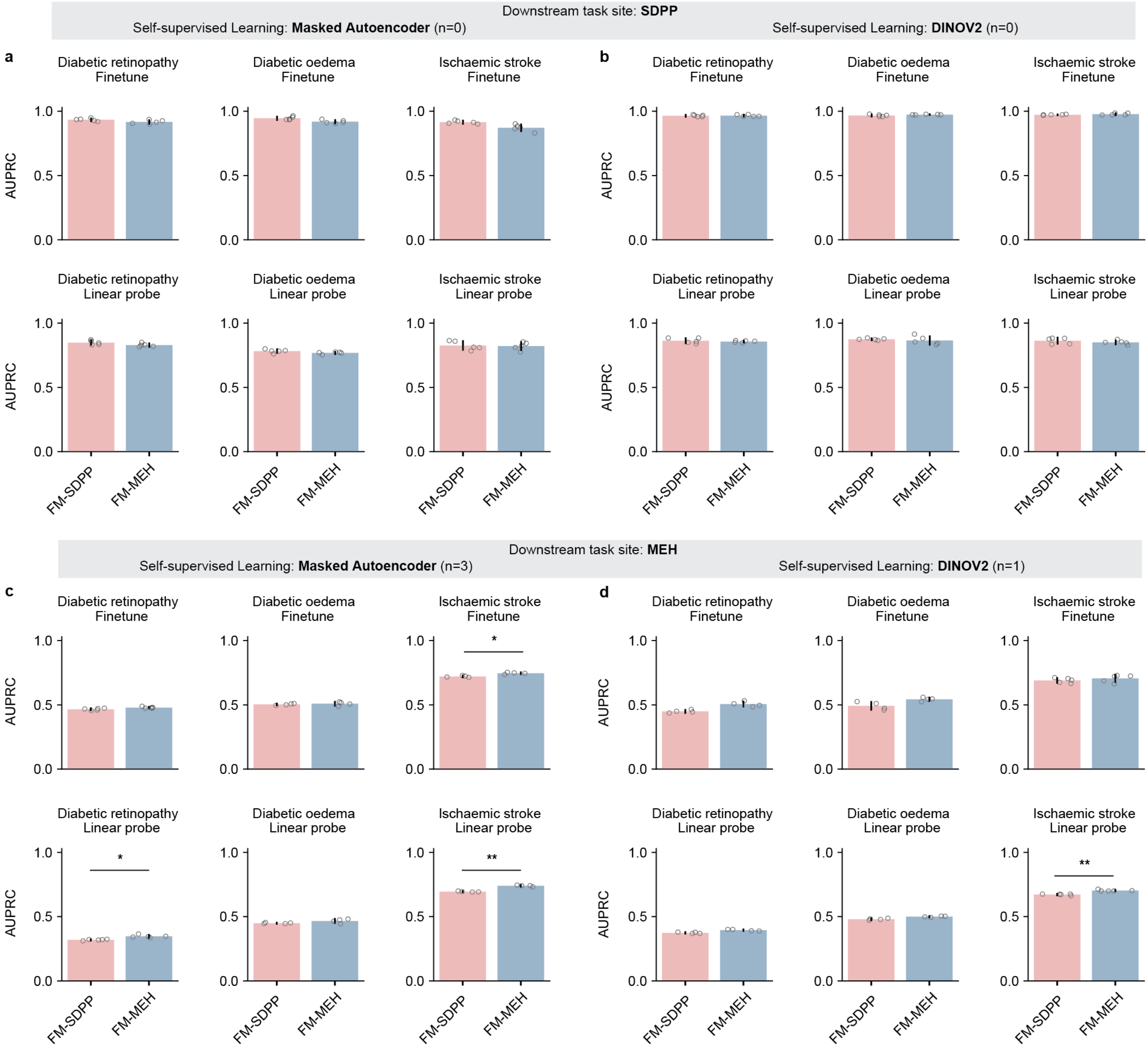

Supplementary Figure 4. AUPRC performance of FM-MEH and FM-SDPP on downstream tasks using data from each site. Subgraphs **a** and **b** show the model performance on tasks at the SDPP site, with FMs respectively pre-trained with Masked Autoencoder and DINOV2. Subgraphs **c** and **d** present the performance of FMs on tasks at the MEH site. In SDPP downstream tasks, FM-SDPP and FM-MEH achieved comparable performance, while FM-MEH achieved superior performance on 4 out of 12 evaluations when adapted to MEH downstream tasks. For each task, models were fine-tuned with five different random seeds, controlling the shuffling of fine-tuning data, and evaluated on the test set to generate five replicates. The mean AUPRC values are represented by bar centres, with error bars indicating 95% CI. A two-sided Welch's t-test followed by Holm-Bonferroni correction (n=24) was used to assess whether the performance differences between FM-SDPP and FM-MEH were statistically significant. * indicates 0.01<p<0.05 and ** indicates p<0.01. *n* indicates the number of cases showing significant differences. All quantitative results, including raw p-value and adjusted p-value, are included in Supplementary Data 3.

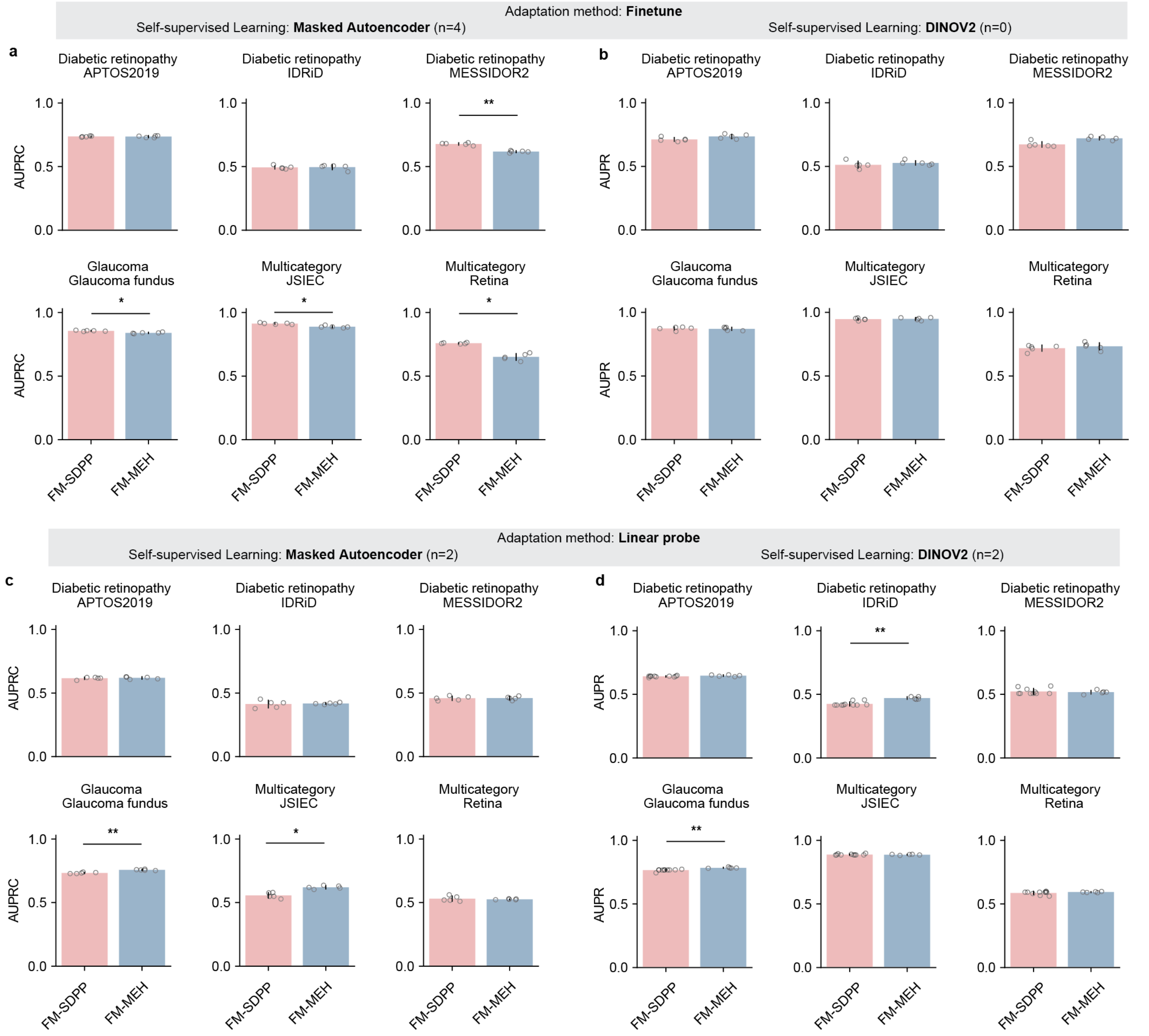

Supplementary Figure 5. AUPRC performance of FM-MEH and FM-SDPP on downstream tasks using publicly available datasets sourced from multiple countries. Subgraphs **a** and **b** show the performance of FMs pre-trained respectively with Masked Autoencoder and DINOV2 when fine-tuned to downstream tasks. Subgraphs **c** and **d** present the performance of FMs when adapted to downstream tasks with the linear probe. When pre-trained with Masked Autoencoder, FM-SDPP significantly outperformed FM-MEH on 4 downstream evaluations. When pre-trained with DINOV2, FM-MEH significantly outperformed FM-SDPP on 2 evaluations. For each task, models were fine-tuned with five different random seeds, controlling the shuffling of fine-tuning data, and evaluated on the test set to generate five replicates. The mean AUROC values are represented by bar centres, with error bars indicating 95% CI. A two-sided Welch's t-test followed by Holm-Bonferroni correction (n=24) was used to assess whether the performance differences between FM-SDPP and FM-MEH were statistically significant. * indicates $0.01<p<0.05$ and ** indicates $p<0.01$. *n* indicates the number of cases showing significant differences. All quantitative results, including raw p-value and adjusted p-value, are included in Supplementary Data 3.

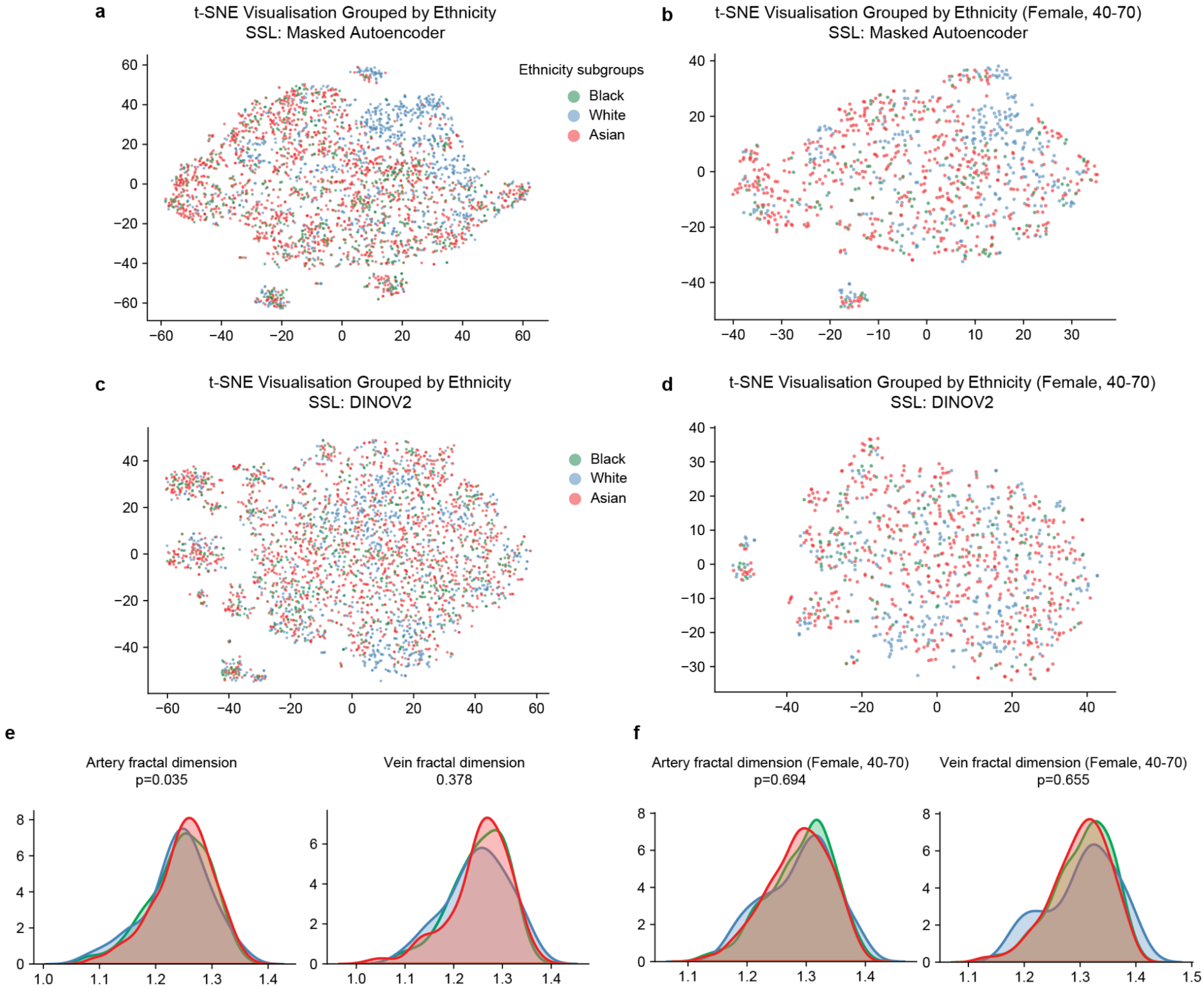

Supplementary Figure 6. Data characteristics of ethnicity subgroups. **a** and **b** show t-SNE visualisation with data features extracted by FM-MEHs pre-trained with Masked Autoencoder and DINOV2, respectively. **c** and **d** show t-SNE visualisation after eliminating confounding effects by specifying sex and age (e.g. Female, 40-70 years old). The ethnicity subgroups show clustering only on **c**. **e** demonstrates the distribution density of clinically meaningful morphological indices over ethnicity subgroups. **f** shows the distribution density after specifying sex and age. A Kruskal-Wallis H-test followed by Holm-Bonferroni correction (n=2) was conducted to assess statistical significance. No significant differences have been observed between subgroups in **f**.

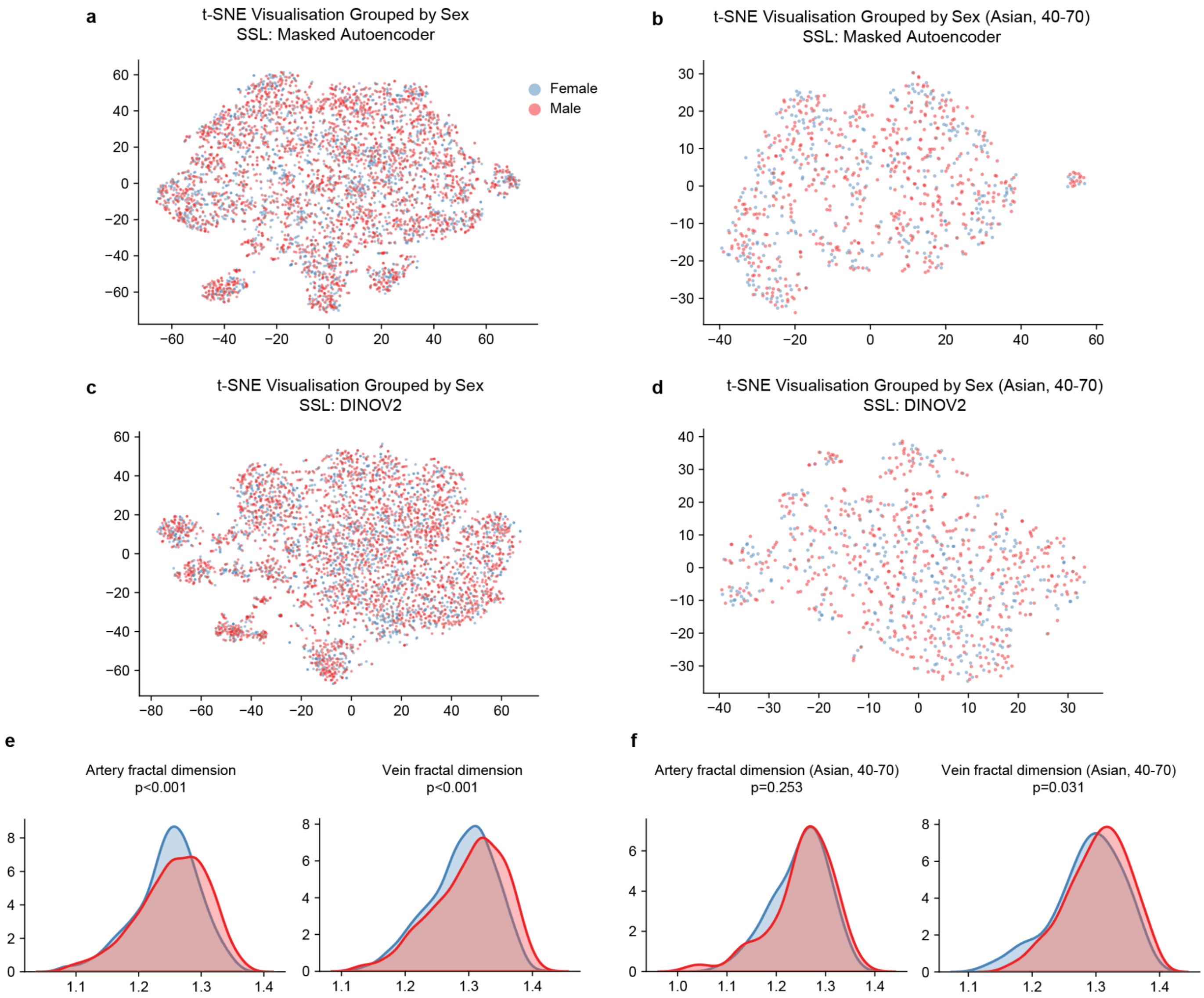

Supplementary Figure 7. Data characteristics of sex subgroups. **a** and **b** show t-SNE visualisation with data features extracted by FM-MEHs pre-trained with Masked Autoencoder and DINOV2, respectively. **c** and **d** show t-SNE visualisation after eliminating confounding effects by specifying ethnicity and age (e.g. Asian, 40-70 years old). The sex subgroups show no distinct clustering. **e** demonstrates the distribution density of clinically meaningful morphological indices over sex subgroups. **f** shows the distribution density after specifying age and ethnicity. A two-sided Welch's t-test followed by Holm-Bonferroni correction (n=2) was conducted to assess statistical significance. Significant differences have been observed in the artery fractal dimension (p=3.19E-4) vein fractal dimension (p=1.87E-5) between sex subgroups, even after controlling the ethnicity and age.

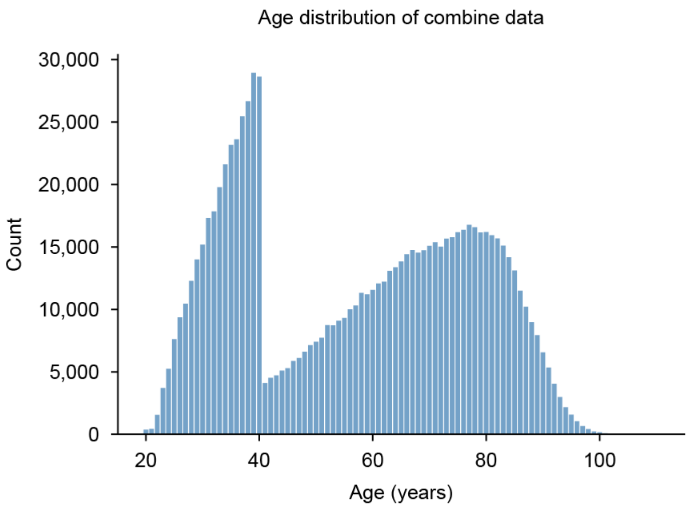

Supplementary Figure 8. Age distribution of the combined dataset, comprising 604,170 real images from MEH and 300,000 synthetic images representative of the younger SDPP cohort (age < 40 years). Pre-training the retinal FM on this combined dataset allows us to examine whether age-related fairness improves, particularly within the younger subgroup in downstream tasks. We note that image synthesis may not always strictly follow the age condition. However, as the SDPP data used for Stable Diffusion XL training are drawn from a young cohort, the resulting synthetic images should still be broadly representative of this group.

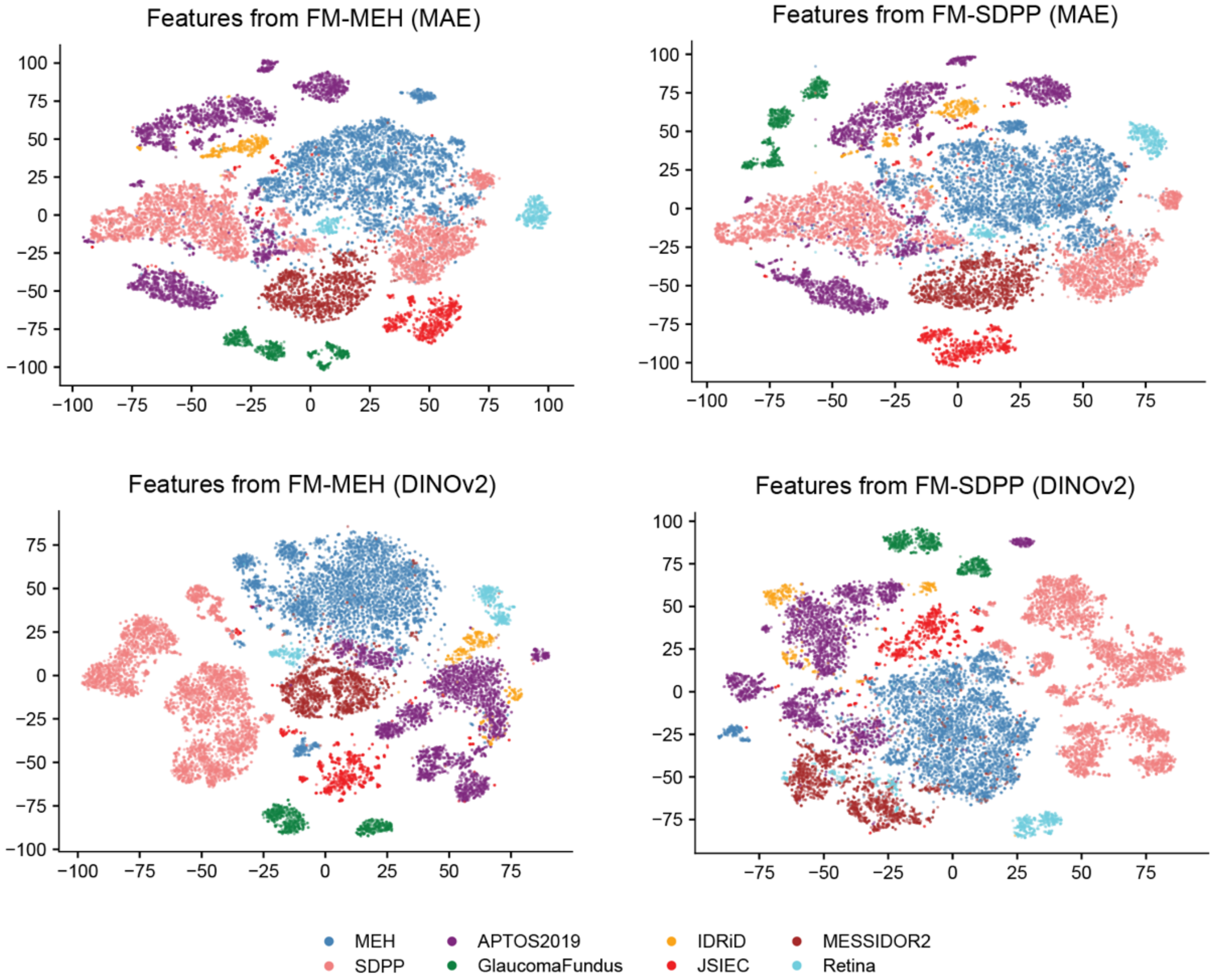

Supplementary Figure 9. t-SNE visualisation for MEH, SDPP data (5000 randomly sampled data points), and publicly available datasets used in this study, respectively with features extracted by foundation models developed in each site (FM-MEH and FM-SDPP) with Masked Autoencoder (MAE) and DINOv2.
