# Supplementary material for: Understanding pre-training data effects in retinal foundation models using two large fundus cohorts
Source: Nat Commun. 2026 Feb 28;17:3309. doi: 10.1038/s41467-026-70077-z (PMC13065816; doi:10.1038/s41467-026-70077-z)
Supplement: Supplementary file 3 — Description of Additional Supplementary Files [file 41467_2026_70077_MOESM3_ESM.pdf]

Description of Additional Supplementary Files

File Name: Supplementary Data 1
Description: Data characteristics of publicly available datasets used in this study.

File Name: Supplementary Data 2
Description: Data characteristics of Moorfields Eye Hospital cohorts and Shanghai cohorts for downstream applications, including diabetic retinopathy detection, diabetic macular oedema detection, and ischaemic stroke prediction.

File Name: Supplementary Data 3
Description: Performance comparison of paralleled foundation models respectively developed using Moorfields Eye Hospital data and Shanghai data.

File Name: Supplementary Data 4
Description: Data characteristics of organised Moorfields Eye Hospital data for fairness evaluation across age, ethnicity, and sex subgroups.

File Name: Supplementary Data 5
Description: Fairness comparison between foundation models developed respectively with Moorfields Eye Hospital data and Shanghai data.

File Name: Supplementary Data 6
Description: Performance comparison of foundation models developed with Masked Autoencoder and DINOv2.

File Name: Supplementary Data 7
Description: Performance comparison between foundation models developed using Moorfields Eye Hospital data, versus data combining Moorfields Eye Hospital samples and synthetic data representative Shanghai young cohorts.

File Name: Supplementary Data 8
Description: Fairness comparison between foundation models developed respectively using Moorfields Eye Hospital data, versus data combining Moorfields Eye Hospital samples and synthetic data representative Shanghai young cohorts.

File Name: Supplementary Data 9
Description: Data distribution distance between the pre-training data and downstream application data.
