# Supplementary material for: Understanding pre-training data effects in retinal foundation models using two large fundus cohorts
Source: Nat Commun. 2026 Feb 28;17:3309. doi: 10.1038/s41467-026-70077-z (PMC13065816; doi:10.1038/s41467-026-70077-z)
Supplement: Supplementary file 5 — Reporting Summary [file 41467_2026_70077_MOESM5_ESM.pdf]

Corresponding author(s): Yukun Zhou, Bin Sheng, Pearse A. Keane

Last updated by author(s): Jan 30, 2026

Reporting Summary

Nature Portfolio wishes to improve the reproducibility of the work that we publish. This form provides structure for consistency and transparency in reporting. For further information on Nature Portfolio policies, see our Editorial Policies and the Editorial Policy Checklist.

Statistics

For all statistical analyses, confirm that the following items are present in the figure legend, table legend, main text, or Methods section.

n/a	Confirmed	
[ ]	[x]	The exact sample size (*n*) for each experimental group/condition, given as a discrete number and unit of measurement
[ ]	[x]	A statement on whether measurements were taken from distinct samples or whether the same sample was measured repeatedly
[ ]	[x]	The statistical test(s) used AND whether they are one- or two-sided<br>*Only common tests should be described solely by name; describe more complex techniques in the Methods section.*
[x]	[ ]	A description of all covariates tested
[ ]	[x]	A description of any assumptions or corrections, such as tests of normality and adjustment for multiple comparisons
[ ]	[x]	A full description of the statistical parameters including central tendency (e.g. means) or other basic estimates (e.g. regression coefficient) AND variation (e.g. standard deviation) or associated estimates of uncertainty (e.g. confidence intervals)
[ ]	[x]	For null hypothesis testing, the test statistic (e.g. *F*, *t*, *r*) with confidence intervals, effect sizes, degrees of freedom and *P* value noted<br>*Give P values as exact values whenever suitable.*
[x]	[ ]	For Bayesian analysis, information on the choice of priors and Markov chain Monte Carlo settings
[x]	[ ]	For hierarchical and complex designs, identification of the appropriate level for tests and full reporting of outcomes
[x]	[ ]	Estimates of effect sizes (e.g. Cohen's *d*, Pearson's *r*), indicating how they were calculated

Our web collection on statistics for biologists contains articles on many of the points above.

Software and code

Policy information about availability of computer code

Data collection	The code used to train, fine-tune and evaluate RETFound from Yukun Zhou is available at https://github.com/rmaphoh/RETFound, which is based on PyTorch. All pre-trained model weights are available at https://huggingface.co/YukunZhou. Images were processed with automated retinal image analysis tool AutoMorph v.1.0 (https://github.com/rmaphoh/AutoMorph).
Data analysis	Data was analysed with Python v3.11.0 (https://www.python.org/), NumPy v1.26.4 (https://github.com/numpy/numpy), SciPy v1.15.2 (https://www.scipy.org/), Matplotlib v3.8.4 (https://github.com/matplotlib/matplotlib), pandas v1.5.0 (https://github.com/pandas-dev/pandas), Scikit-Learn v1.4.2 (https://scikit-learn.org/stable), Pillow v10.2.0 (https://pypi.org/project/Pillow).

For manuscripts utilizing custom algorithms or software that are central to the research but not yet described in published literature, software must be made available to editors and reviewers. We strongly encourage code deposition in a community repository (e.g. GitHub). See the Nature Portfolio guidelines for submitting code & software for further information.

Data

Policy information about availability of data

All manuscripts must include a data availability statement. This statement should provide the following information, where applicable:

- Accession codes, unique identifiers, or web links for publicly available datasets
- A description of any restrictions on data availability
- For clinical datasets or third party data, please ensure that the statement adheres to our policy

The MEH data consists of routinely collected healthcare data. Owing to their sensitive nature, the dataset is subject to controlled access by means of a structured application process. The AlzEye dataset is subject to the contractual restrictions of the data sharing agreements between National Health Service Digital, Moorfields Eye Hospital and University College London due to data privacy and ethical requirement. Interested collaborators should contact the chief investigator P.A.K at p.keane@ucl.ac.uk and submit a formal submission at https://www.insight.hdrhub.org/insight-data. The data management committee will then review all the requests and grants. A formal data transfer agreement will be required upon approval. Generally, all these requests for access to the data will be responded to within 1month.
For SDPP data, due to data privacy and ethical requirement, individual-level patient data can be accessible with the consent of the data management committee from institutions and are not publicly available. Requests for the non-profit use of the fundus images and related clinical information should be sent to T.Y.W at wongtienyin@tsinghua.edu.cn. The data management committee will then review all the requests and grants. A formal data transfer agreement will be required upon approval. The requests for access to the data will be responded to within 1month.

Data for ocular disease experiments are publicly available online and can be accessed through the following links: IDRID (https://ieee-dataport.org/open-access/indian-diabetic-retinopathy-image-dataset-idrid), MESSIDOR2 (https://www.adcis.net/en/third-party/messidor2/), APTOS2019 (https://www.kaggle.com/competitions/aptos2019-blindness-detection/data), Glaucoma Fundus (https://dataverse.harvard.edu/dataset.xhtml?persistentId=doi:10.7910/DVN/1YRRAC), JSIEC (https://zenodo.org/record/3477553), and Retina (https://www.kaggle.com/datasets/jr2ngb/cataractdataset).

Research involving human participants, their data, or biological material

Policy information about studies with human participants or human data. See also policy information about sex, gender (identity/presentation), and sexual orientation and race, ethnicity and racism.

Reporting on sex and gender	Biological sex information for MEH-AlzEye and Shanghai-SDPP was collected via self-report. MEH-AlzEye includes 353,157 patients (190,494 female and 162,663 male). Shanghai-SDPP includes 79,284 participants. Experiments were conducted both on female and male. We used part of MEH-AlzEye and Shanghai-SDPP data to develop parallel foundation models, respectively, and held-out data for downstream validation (detailed in Supplementary Table 2).
Reporting on race, ethnicity, or other socially relevant groupings	Ethnicity information for MEH-AlzEye and Shanghai-SDPP was collected via self-report. The MEH-AlzEye data used in this study includes White (45.9%), Asian or Asian British (18.7%, of which 0.6% were Chinese), Black or Black British (9.2%), Mixed (0.9%), other ethnicity (12.7%) and not reported (NR, 12.6%), based on the ethnicity grouping by the UK Office for National Statistics. The SDPP data used in this study includes Chinese (100%).
Population characteristics	MEH-AlzEye is a retrospective cohort study linking ophthalmic data of 353,157 patients, who attended Moorfields Eye Hospital between 2008 and 2018, with systemic health data from hospital admissions across the whole of England. Systemic health data are derived from Hospital Episode Statistics (HES) data relating to admitted patient care (APC), with a focus on cardiovascular disease and all-cause dementia. More details can be found in the method section. SDPP cohort was drawn from a community-based longitudinal study of 79,284 participants who underwent physical examinations between December 2015 and November 2022. The average age in the MEH cohort is 68.88 years (95% Confidence Interval (CI) 68.85, 68.91), significantly older than the SDPP cohort, which had an average age of 47.26 years (95% CI 47.21, 47.30) (P<0.001). MEH data had a more balanced sex distribution, with 52.8% female participants compared to 36.8% in the SDPP data.
Recruitment	MEH-AlzEye is a retrospective cohort study linking ophthalmic data of 353,157 patients who attended Moorfields Eye Hospital between 2008 and 2018. Shanghai-SDPP is a retrospective cohort study linking ophthalmic data of 79,284 participants who underwent physical examinations at Huadong Sanatorium and Shanghai Sixth People's Hospital between December 2015 and November 2022
Ethics oversight	This study involves human participants and was approved by the London-Central Research Ethics Committee (18/LO/1163, approved 1 August 2018), Advanced statistical modelling of multimodal data of genetic and acquired retinal diseases (20/HRA/2158, approved 5 May 2020), Confidential Advisory Group for Section 251 support (18/CAG/0111, approved 13 September 2018), and the Ethics Committee of Shanghai Sixth People's Hospital (Approved No: 2019-087, approved 29 August 2019). The National Health Service Health Research Authority gave final approval on 13 September 2018. Moorfields Eye Hospital NHS Foundation Trust validated the de-identifications for MEH data. Only de-identified retrospective data were used for research.

Note that full information on the approval of the study protocol must also be provided in the manuscript.

Field-specific reporting

[illegible]

Life sciences study design

All studies must disclose on these points even when the disclosure is negative.

Sample size	Data for developing parallel foundation models was sampled from MEH-AlzEye and Shanghai-SDPP (each contributing 904,170 CFPs). Data for ocular disease diagnosis were from public datasets, detailed in Supplementary Table 1. Data for comparing intra-site and inter-site adaptation were from held-out MEH-AlzEye and held-out Shanghai-SDPP, introduced in Supplementary Table 2. Datasets were chosen based on the availability of labels that would permit model comparison, which is dependent on the specific clinical task being evaluated. The chosen validation datasets were deemed to be suitable based on their parameters, which are summarised Supplementary Table 1 Dataset characteristics. Formal sample size calculations were not performed due to the lack of established methods when applied to machine-learning classification studies.
Data exclusions	Data failed image processing with AutoMorph were excluded. Data without systemic health labels were excluded. For more details please refer to the method section.
Replication	All patients were randomly selected and were not correlated in any way. The replication of experiment results were confirmed in 5 times with 5 different random seeds.
Randomization	The training/validation/testing data for downstream tasks were randomly splitted. For each patient, we only included one eye data from one visit to avoid potential bias by inconsistent individual visits.
Blinding	When assigning patients randomly to training, validation and testing groups investigators were blinded to patient covariates and all features in the dataset not required to perform the research.

Reporting for specific materials, systems and methods

We require information from authors about some types of materials, experimental systems and methods used in many studies. Here, indicate whether each material, system or method listed is relevant to your study. If you are not sure if a list item applies to your research, read the appropriate section before selecting a response.

Materials & experimental systems

n/a	Involved in the study
☒	☐ Antibodies
☒	☐ Eukaryotic cell lines
☒	☐ Palaeontology and archaeology
☒	☐ Animals and other organisms
☒	☐ Clinical data
☒	☐ Dual use research of concern
☒	☐ Plants

Methods

n/a	Involved in the study
☒	☐ ChIP-seq
☒	☐ Flow cytometry
☒	☐ MRI-based neuroimaging

Plants

Seed stocks	*Report on the source of all seed stocks or other plant material used. If applicable, state the seed stock centre and catalogue number. If plant specimens were collected from the field, describe the collection location, date and sampling procedures.*
Novel plant genotypes	*Describe the methods by which all novel plant genotypes were produced. This includes those generated by transgenic approaches, gene editing, chemical/radiation-based mutagenesis and hybridization. For transgenic lines, describe the transformation method, the number of independent lines analyzed and the generation upon which experiments were performed. For gene-edited lines, describe the editor used, the endogenous sequence targeted for editing, the targeting guide RNA sequence (if applicable) and how the editor was applied.*
Authentication	*Describe any authentication procedures for each seed stock used or novel genotype generated. Describe any experiments used to assess the effect of a mutation and, where applicable, how potential secondary effects (e.g. second site T-DNA insertions, mosiacism, off-target gene editing) were examined.*
